# Age and sex as confounding factors in the relationship between cardiac mitochondrial function and type 2 diabetes in the Nile Grass rat

**DOI:** 10.1371/journal.pone.0228710

**Published:** 2020-02-21

**Authors:** Jillian Schneider, Woo Hyun Han, Rebecca Matthew, Yves Sauvé, Hélène Lemieux

**Affiliations:** 1 Faculty Saint-Jean, University of Alberta, Edmonton, Alberta, Canada; 2 Department of Physiology, University of Alberta, Edmonton, Alberta, Canada; 3 Department of Ophthalmology and Visual Sciences, University of Alberta, Edmonton, Alberta, Canada; 4 Department of Medicine, Women and Children's Health Research Institute, University of Alberta, Edmonton, Alberta, Canada; Universidade do Estado do Rio de Janeiro, BRAZIL

## Abstract

Our study revisits the role of cardiac mitochondrial adjustments during the progression of type 2 diabetes mellitus (T2DM), while considering age and sex as potential confounding factors. We used the Nile Grass rats (NRs) as the animal model. After weaning, animals were fed either a Standard Rodent Chow Diet (SRCD group) or a Mazuri Chinchilla Diet (MCD group) consisting of high-fiber and low-fat content. Both males and females in the SRCD group, exhibited increased body mass, body mass index, and plasma insulin compared to the MCD group animals. However, the females were able to preserve their fasting blood glucose throughout the age range on both diets, while the males showed significant hyperglycemia starting at 6 months in the SRCD group. In the males, a higher citrate synthase activity—a marker of mitochondrial content—was measured at 2 months in the SRCD compared to the MCD group, and this was followed by a decline with age in the SRCD group only. In contrast, females preserved their mitochondrial content throughout the age range. In the males exclusively, the complex IV capacity expressed independently of mitochondrial content varied with age in a diet-specific pattern; the capacity was elevated at 2 months in the SRCD group, and at 6 months in the MCD group. In addition, females, but not males, were able to adjust their capacity to oxidize long-chain fatty acid in accordance with the fat content of the diet. Our results show clear sexual dimorphism in the variation of mitochondrial content and oxidative phosphorylation capacity with diet and age. The SRCD not only leads to T2DM but also exacerbates age-related cardiac mitochondrial defects. These observations, specific to male NRs, might reflect deleterious dietary-induced changes on their metabolism making them more prone to the cardiovascular consequences of aging and T2DM.

## Introduction

Aging is a predominant risk factor for type 2 diabetes (T2DM) and related cardiovascular disease [1, 2]. The worldwide incidence of T2DM has increased from 108 to 422 million individuals between 1980 and 2014, and the upward trend persists [3]. Cardiovascular complications are the number one cause of mortality in T2DM patients; the complications include accelerated atherosclerosis, systolic and diastolic dysfunction, left ventricular hypertrophy, atrial fibrosis and fibrillation, heart failure, and increased post myocardial infarction fatality [4]. In cardiomyocytes, insulin and nutritional signaling operate as major regulators of metabolism, ensuring the maintenance of appropriate glucose response, lipidemia, oxidative stress, inflammation, calcium handling, apoptosis, and mitochondrial function [5]. A salient role of mitochondria in T2DM is supported by findings that mitigation of insulin resistance also improves mitochondrial health [6].

The human heart needs a substantial amount of ATP to beat approximately 100,000 times daily. About 95% of ATP consumed by cardiac muscle comes from the oxidative phosphorylation (OXPHOS) process located within the mitochondria [7]. Electrons travel through the electron transfer pathways (ET-pathways) in the inner mitochondrial membrane while protons are pumped into the intermembrane space; this creates a proton gradient used by ATP synthase to phosphorylate ADP into ATP. In healthy cardiomyocytes, over 60–70% of ATP is supplied from fatty acids as electron donors; the remaining electrons are obtained from the oxidation of glucose, lactate, amino acids, or ketone bodies [7]. Oxygen acts as the final electron acceptor. Electrons leaking from the ET-pathways can partially reduce oxygen, producing reactive oxygen species (ROS). Changes in mitochondrial metabolism will not only affect the energetic status but also the production of ROS; these will have a concurrent impact on cellular aging.

Cardiac mitochondrial defects have been described in T2DM (reviewed by [8]); the reported changes are in the capacity/activity/content of various mitochondrial ET-pathway complexes [9–15], pyruvate dehydrogenase complex (PDC) activity [16–18], coupling [15, 19], oxidative stress [20, 21], mitochondrial content [9, 17], mitochondrial respiration supported by fatty acid substrates [9, 13, 17, 22–24], and mitochondrial network fragmentation [19]. The results in the literature mostly represent a single time point, after hyperglycemia onset and/or in models of rapid T2DM progression. In humans, diabetes is a slowly evolving disease, with its occurrence increasing 2.5-fold after 65 years of age [25]. As aging progresses, mitochondrial dysfunctions arising from T2DM become confounded by those attributed to aging alone [26–28], complicating our understanding of the role of mitochondrial dysfunction in T2DM.

The Nile Grass rat (NR), *Arvicanthis niloticus*, mimics the slow progression of T2DM and related complications observed in human [29–31]. An asset of this animal model is that no pharmacological agent or genetic manipulation is needed to induce the disease. When fed a Standard Rodent Chow Diet (SRCD group), males spontaneously exhibit pathological markers congruent with metabolic syndrome and T2DM [29, 32, 33]. At 2 months, the males fed the SRCD show insulin resistance and hyperinsulinemia, which progress toward hyperglycemia at 6 months. On a high-fiber low-fat diet (Mazuri Chinchilla Diet, MCD group), NRs maintain normoglycemia throughout their lifespan [29]. Furthermore, the males fed a SRCD have a higher susceptibility to develop subtle diastolic cardiac dysfunction [34]. The females have been studied less, but studies show they are able to preserve their fasting blood glucose (FBG) and cardiovascular function for at least 12 months on a SRCD [34]. The consideration of sexual dimorphism might be a critical and neglected factor in our understanding T2DM. In humans, although the prevalent statistics show the same percentage for males and females (9.4% and 8.9%, respectively), a higher mortality rate is detected in males (54.2% in males versus 45.8% in females) [35]. Furthermore, males are mostly undiagnosed, particularly for hyperinsulinemia (40.2% in males versus 27.8% in females) [35]. A better resistance to diabetes was measured in the females, compared to the males, when the rats were fed a high-fat diet. This was associated with sexual dimorphism in mitochondrial function and resistance to oxidative stress [36]. A New Zealand obese mouse model of T2DM also showed that both sexes develop obesity when on a high fat diet with carbohydrates, but only male mice manifest T2DM and cardiac mitochondrial dysfunction [37]. The sexual dimorphism has been overlooked in many studies focusing on males.

Our study wants to address three neglected factors that interfere with our understanding of the role of cardiac mitochondrial dysfunction in T2DM. The first one is addressing the early stages of the disease, before hyperglycemia. The second one is the complex and continuous aging processes that occur concurrently with the disease and the influence on the mitochondria. The third one is the sexual dimorphism associated not only with T2DM, but also with aging [38, 39] and cardiovascular disease [40]. We suspect that addressing these aspects all together might be the key to delineate the role of mitochondria in cardiovascular pathologies associated with T2DM and aging. By including females in our experimental design, we will also study the effects of a MCD on preventing cardiac mitochondrial damage, both in the presence or in the absence of hyperglycemia.

## Material and methods

### Animals

Nile Grass rats (*Arvicanthis niloticus*) were maintained on a 14:10 h light-dark cycle, at 21±2°C room temperature and ~40% relative humidity. After weaning at 21–23 days, the males and females were each divided into two dietary groups: 1) MCD group, fed Mazuri® Chinchilla (5M01, Purina Mills, LLC, St. Louis, MO, USA; 4.0% fat, 15.3% fibers, 21.6% protein); 2) SRCD group fed Prolab® (RMH 2000, 5P06, LabDiet, Nutrition Intl., Richmond, IN, USA; 9.6% fat, 3.2% fibers, 19.9% protein). The animals received food and water *ad libitum*. The metabolic phenotype and cardiac mitochondrial function were measured at 2, 6 and 18 months of biological age. A fasting period of 16–18 h preceded blood and tissue collection. All experiments were approved by the University of Alberta Institutional Animal Care and Use Committee (protocol 328) and the NIH (USA) guidelines regarding the care and use of animals for experimental procedures.

### Metabolic phenotype

Animals were euthanized with intraperitoneal injection of 480 mg kg^-1^ of Euthanyl (Bimeda-MTC Animal Health, Inc., Cambridge, ON, Canada). Animals were then weighted, measured, and their fasting plasma insulin and fasting blood glucose (FBG) levels were assessed as described previously [41]. Fasting insulin levels > 2 ng ml^-1^ indicated compensation and FBG > 5.0 mmol l^-1^ reflected hyperglycemia [29].

### Cardiac permeabilized fiber preparation

Whole heart was collected immediately after euthanasia and immersed in ice-cold muscle relaxing solution BIOPS [42]. Approximately 30 mg of left ventricular tissue from the apex was transferred into fresh ice-cold relaxing solution. The fibers were mechanically permeabilized with forceps, followed by gentle agitation for 30 min at 4°C in relaxing solution supplemented with 50 μg ml^-1^ saponin [43]. Fibers were washed for 10 min by agitation in ice-cold mitochondrial respiration medium MiR05 [44]. The fibers were then blotted, weighted, and immediately used for respirometric measurements.

### High-resolution respirometry

Respirometric measurements were performed at 37°C using 2.0–4.5 mg of permeabilized ventricular fibers per chamber of the Oxygraph-2K (Oroboros Instruments, Innsbruck, Austria) filled with 2 ml of MiR05. Datlab software (Oroboros Instruments, Innsbruck, Austria) was used for data acquisition and analysis. Artificial oxygen diffusion limitation was avoided by maintaining oxygen levels over 200 μM O_2_ [43], and instrumental oxygen background fluxes were calibrated as a function of oxygen concentration and subtracted from the total volume-specific oxygen flux using Datlab software (Oroboros Instruments). Four protocols were applied to the permeabilized fibers to evaluate the capacity of electron transfer (ET) pathways and steps (protocol 1) or fatty acid β-oxidation (Protocols 2, 3, 4; Table 1). Two states were included: (1) LEAK respiration in the non-phosphorylated state without ADP, and (2) OXPHOS capacity coupled to phosphorylation of ADP to ATP in the presence of saturating ADP. The first protocol includes the measurement of LEAK respiration (in the presence of 5 mM pyruvate and 5 mM malate), OXPHOS capacity for the NADH pathway (N-pathway, flux through complex I in the presence of pyruvate, malate, and 2.5 mM ADP), the NADH & Succinate pathway (NS-pathway, convergent electron flux through complex I and II in the presence of pyruvate, malate, 10 mM succinate, and ADP), the Succinate pathway (S-pathway, electron flux through complex II after inhibition of complex I with 1 μM rotenone), the residual oxygen consumption (ROX) after inhibition of complex III with 5 μM antimycin A, the complex IV activity (with 2 mM ascorbate and 0.5 mM tetramethylphenylenediamine (TMPD), feeding electrons into complex IV), and the chemical background with 100 mM sodium azide (substracted from complex IV capacity). A variation of protocol 1, including the addition of the uncoupler dinitrophenol (DNP) after succinate was performed on a reduced number of animals to confirm no limitation of the OXPHOS capacity by the phosphorylation system in cardiac fibers from the NRs, as previously measured in the rat [27] and mouse [43] hearts. The following three protocols included the measurement of LEAK respiration and OXPHOS capacity in the presence of three different fatty acids substrates combinations, i.e., 0.04 mM palmitoylcarnitine and 5 mM malate, 0.20 mM octanoylcarnitine and malate, as well as 5 mM acetylcarnitine and malate. In each protocol, 10 μM cytochrome *c* was added after ADP to test for integrity of outer mitochondrial membrane; an increase of respiration of more than 20% was estimated as an exclusion criteria and was present in only two experiments.

**Table 1:** Evaluation of mitochondrial oxidative phosphorylation capacity in the presence of different substrate combinations, and the related pathways and specific steps targeted by the measurement.

Protocols	Substrate combinations (with saturating ADP and cytochrome *c*)	Pathways	Specific complexes, enzymes, and transporters measured
1	Pyruvate+malate (PM)	NADH	Complex I
			Pyruvate dehydrogenase complex
			Pyruvate transporter
1	Pyruvate+malate+succinate (PMS)	NADH & Succinate	Complexes I & II
			Pyruvate dehydrogenase complex
			Pyruvate transporter
			Succinate dehydrogenase
1	Succinate+rotenone	Succinate	Complexes II
			Succinate dehydrogenase
1	Ascorbate+TMPD (-azide background)	-	Complex IV single step
2	Palmitoylcarnitine+malate	ETF	Long-chain fatty acid oxidation
			Carnitine translocase
			Carnitine palmitoyltransferase-II
3	Octanoylcarnitine+malate	ETF	Medium-chain fatty acid oxidation
			Carnitine translocase
			Carnitine palmitoyltransferase-II
4	Acetylcarnitine+malate	ETF	Carnitine acetyltransferase
			Carnitine translocase

ETF, electron transferring flavoprotein; TMPD, *N*,*N*,*N*,*N*-tetramethyl-*p*-phenylenediamine

Data of mitochondrial function are presented either as oxygen flux per mass (for all protocols), or as Flux Control Ratios (FCR), normalized for maximal OXPHOS capacity in the presence of substrates feeding electrons simultaneously into the NADH and Succinate pathways (for protocol 1). At the end of each experimental run, the content of the chamber was removed and the chamber was rinsed twice with 500 μL of MiR05. The fibers were homogenized 2 times, 30 s on ice, with glass potters and immediately stored at -80°C for measurement of citrate synthase (CS) as previously described [41].

### Data analysis

Statistical analyses were performed with SigmaPlot 14 (Systat Sofware Inc., San Jose, CA, USA). Graphics were produced using GraphPad Prism 7 (GraphPad Software, Inc., La Jolla California). The criteria of normality and homogeneity of variance for ANOVA were tested for each variable using Kolmogorov-Smirnov (Lilliefor’s correction) and Brow-Forsythe tests, respectively. The data of mitochondrial function and metabolic phenotype were then analyzed using three-way ANOVA with sex, diet, and age as the three factors, and was followed by pairwise Tukey’s tests. Some variables were transformed to meet the criteria of the ANOVA and the specific transformation are indicated on each figure legend. Data are presented as median (min–max) without transformation, were N is the number of animals, and n is the number of oxygraph assays. A p<0.05 was considered significant. The data underlying this study have been deposited to Dryad and may be accessed via doi:10.5061/dryad.0rp87td.

## Results

### Both males and females display obesity associated with the SRCD diet, but only females are able to preserve fasting blood glucose in the normal range

**Fig 1:**
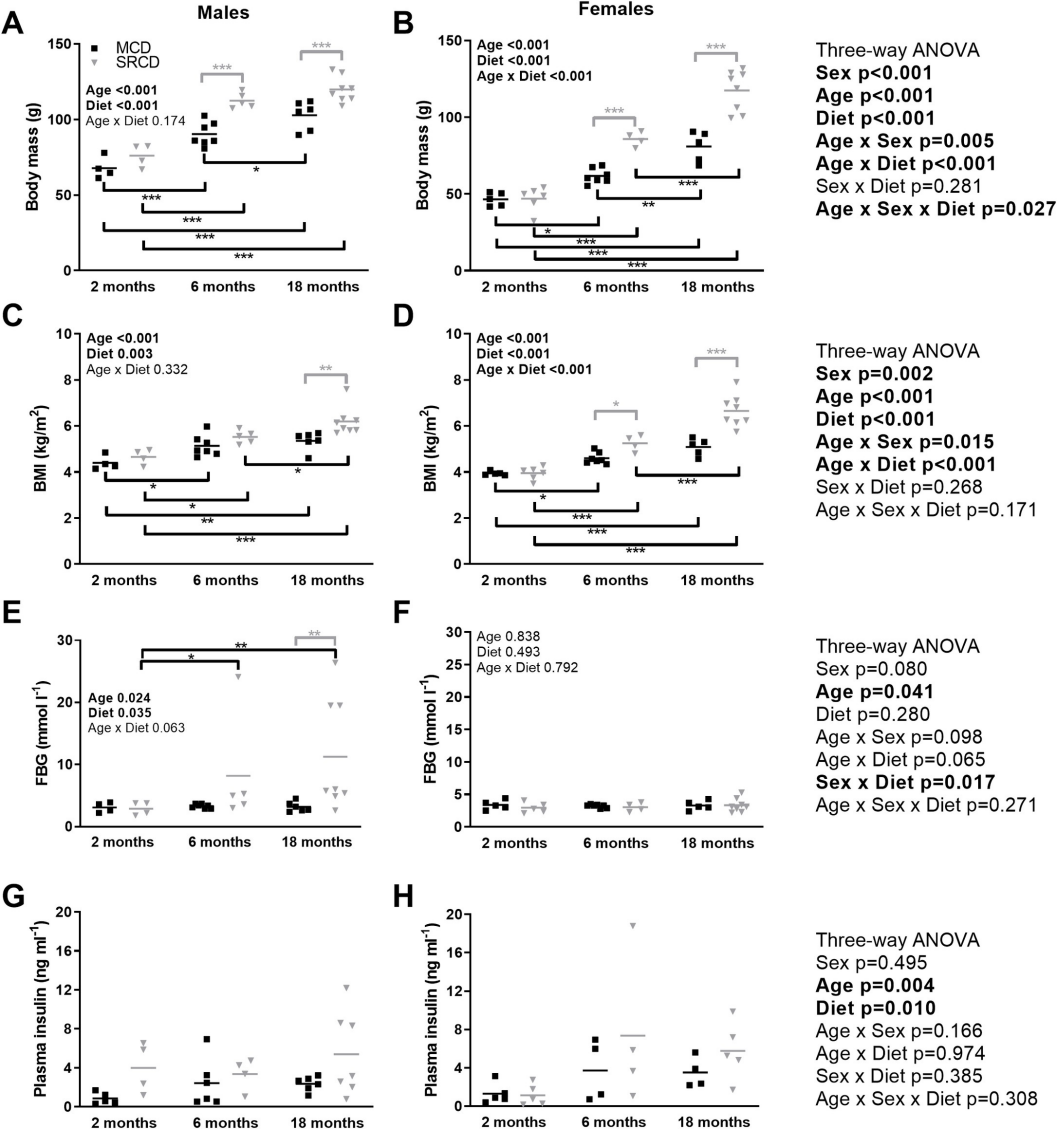
Nile Grass rat metabolic profiles throughout the age range. Metabolic phenotype of the males (left side) and females (right side) Nile Grass rats fed the Mazuri Chinchilla Diet (MCD group, black squares) or the Standard Rodent Chow Diet (SRCD, grey triangles), at three different ages (2 months, 6 months, and 18 months). The parameters measured are the body mass (A, B), the body mass index (BMI; B, C), the Fasting Blood Glucose (FBG; E, F) and the Plasma Insulin (G, H). Dot plot indicates the individual data points and the median. Significant differences between dietary (in grey) or age (in black) groups are indicated with *** for p<0.001, ** for p<0.01, * for p<0.05. N = 4–8 animals per age and diet group. The following transformations were applied to meet the assumption of the ANOVAs: square root BMI and plasma insulin, reciprocal for FBG. Data are presented without transformation. Results of the three-way ANOVAs are on the right. When there was a significant effect or interaction with sex, two-way ANOVAs were performed on separate sexes and the results are indicated on individual panels.

For each age point, according to the NR metabolic profiles (Fig 1), the body weight of males fed on the SRCD was higher compared to that of those fed on the MCD; however, it reached significance only at 6 and 18 months (Fig 1A). Similar results were obtained in the females, but with stronger separation between dietary groups at 2 and 18 months of age (Fig 1B). The body mass index (BMI) (Fig 1C and 1D) represents a better indicator of obesity comparison between males and females as it takes into account the smaller length of the females. The BMI was also higher in the SRCD group. The significance between dietary groups was reached at 18 months of age in the males (Fig 1C) and 6 and 18 months of age in the females (Fig 1D). There was no interaction between sex and diet for the BMI (p = 0.268), indicating similar effect of diets in both sexes.

Overall, the only metabolic profile parameter showing a significant interaction between sex and diet in the three-way ANOVA is the FBG (p = 0.017, Fig 1E and 1F, right side). In the males, the FBG was higher in the SRCD group, compared to the MCD group at 6 and 18 months, whereas the significance was only obtained at 18 months due to high variability in the SRCD group (Fig 1E). In contrast, the females preserved their FBG at the normal level throughout the age range (Fig 1F). The plasma insulin was significantly affected by age (p = 0.027 between 2 and 6 months; p = 0.005 between 2 and 18 months; Tuckey pairwise comparisons), and diet (p = 0.010 between MCD and SRCD group; Tuckey pairwise comparison), but not by sex (p = 0.495). Furthermore, there was no significant interaction of any of the factors for plasma insulin levels. We also performed regression analysis between the BM and the insulin level, showing a weak but significant correlation in both sexes (R^2^ = 0.216 and 0.317, in males and females respectively).

### Citrate synthase activity is affected by age and diet, but only in the males

**Fig 2:**
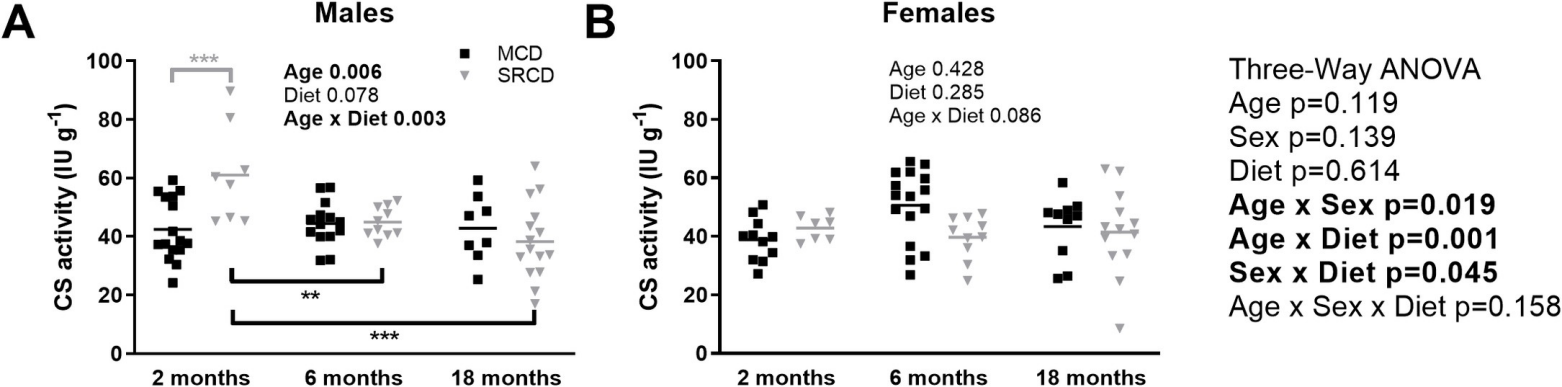
Cardiac citrate synthase activity in the Nile Grass rat throughout the age range. Citrate synthase (CS) activity in cardiac fibers of the males (A) and females (B) Nile Grass rats fed the Mazuri Chinchilla Diet (MCD group, black squares) and the Standard Rodent Chow Diet (SRCD, grey triangles), at three different ages (2 months, 6 months, and 18 months). Data are presented as box and whisker plots of mean CS activity (IU g^-1^ cardiac fibers). The graph on the left is for the male data and on the right for the female data. Dot plot indicates the individual data points and the median. N = 4–12 animals per group, and n = 7–16 fiber preparations per group. Results of the three-way ANOVAs are on the right, and two-way ANOVAs on each panel. Significant differences between dietary groups (on the top, grey) and between age groups (on the bottom, black) are indicated with *** for p<0.001, ** for p<0.01, * for p<0.05.

CS activity was used as a marker of cardiac ventricular mitochondrial content. The three-way ANOVA showed a significant interaction between age and sex (p = 0.019), age and diet (p = 0.001), and sex and diet (p = 0.045; Fig 2, right side). At 2 months of age, the males in the SRCD group started with a higher CS activity compared to age-matched MCD group (p<0.001; Fig 2A). Only the males in the SRCD group (p<0.001) had a significant decrease in mitochondrial content with age compared to the MCD group (p = 0.875; Fig 2A). In contrast, the females showed no significant effect of age or diet on CS activity (Fig 2B).

### The relative capacity of the NADH pathway decreases with age, independent of the sex or diet

**Fig 3:**
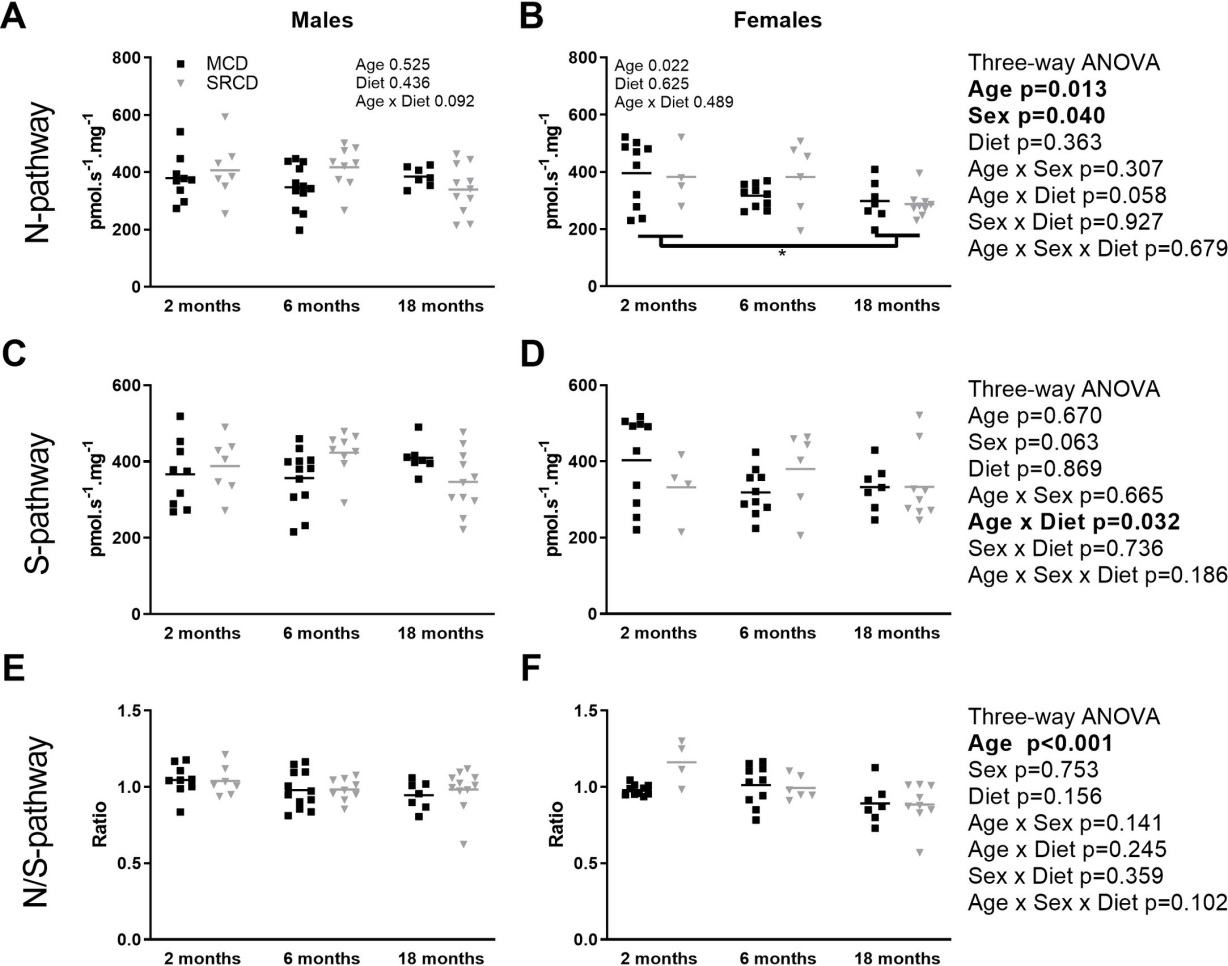
Oxidative phosphorylation capacity in the heart of Nile Grass rat throughout the age range. Oxidative phosphorylation (OXPHOS) capacity in cardiac permeabilized fibers from males (left) or females (right) Nile Grass rats fed the Mazuri Chinchilla Diet (MCD group, black squares) and the Standard Rodent Chow Diet (SRCD, grey triangles), at three ages (2 months, 6 months, and 18 months). Respiratory capacity was expressed in flux per fiber mass (pmol s^-1^ mg^-1^ wet weight) for the NADH pathway (N-pathway; OXPHOS state in the presence of pyruvate, malate, saturating ADP, cytochrome *c*; panels A and B), the Succinate pathway (S-pathway; ET state in the presence of pyruvate, malate, ADP, cytochrome *c*, succinate, rotenone; panels C and D), as well as the ratio of the N/S-pathway (panels E and F). Dot plot indicates the individual data points and the median. The square root transformations were applied to meet the assumption of the female two-way ANOVAs for the N-pathway. Data are presented without transformation. N = 3–7 animals per group, and n = 4–12 fiber preparations per group. Results of the three-way ANOVAs are on the right and two-way ANOVAs on each panel. Significant differences between dietary groups (on the top, grey) and between age groups (on the bottom, black) are indicated with *** for p<0.001, ** for p<0.01, * for p<0.05.

The OXPHOS capacity was measured for the NADH pathway (in the presence of pyruvate and malate, with electrons entering complex I) and for the Succinate pathway (in the presence of succinate and rotenone, with electrons entering complex II). The three-way ANOVA for the OXPHOS capacity of the NADH pathway in flux per mass showed effects of age and sex (Fig 3A and 3B; right side), but the age effect reached significance only for the female (p = 0.022) and not for the males (p = 0.525) in the two-way ANOVA. In contrast, the Succinate pathway, expressed in flux per mass, showed no significant changes with age, sex, or diet (Fig 3C and 3D). However, there was a significant interaction of age and diet shown by an increase in capacity in the SRCD group compared to the MCD group at 6 months (p = 0.022, sexes confounded). The increase in mitochondrial content at 2 months in the males fed the SRCD compared to the males fed the MCD (Fig 2A) did not translate into a higher capacity in flux per mass for the NADH or the Succinate pathway (Fig 3A and 3C).

The OXPHOS capacity was then expressed as NADH/Succinate pathways (N/S-pathways) ratio (Fig 3E and 3F) or as FCRs (results not shown), normalized for maximal OXPHOS capacity (here equivalent to ET capacity) in the presence of substrates feeding electrons into N/S-pathway simultaneously. These ratios represent the proportional contribution of a specific pathway, and therefore are dictated by the mitochondrial properties rather than the mitochondrial content. When expressed as a N/S-pathways ratio (Fig 3E and 3F), the aging difference observed for the NADH pathway alone become stronger (p<0.001) and was unrelated to sex or diet (Fig 3E and 3F). The same results are obtained when the NADH pathway is normalized to FCR with a significant effect of age (p = 0.002; between 2 and 18 months of age), independent of sex (p = 0.710) or diet (p = 0.665). The Succinate pathway, when expressed as FCR, showed no effect of age (p = 0.369), sex (p = 0.127), or diet (p = 0.232), as well as no interaction between the factors.

### Complex IV relative capacity varied with age and diet, but only in the males

**Fig 4:**
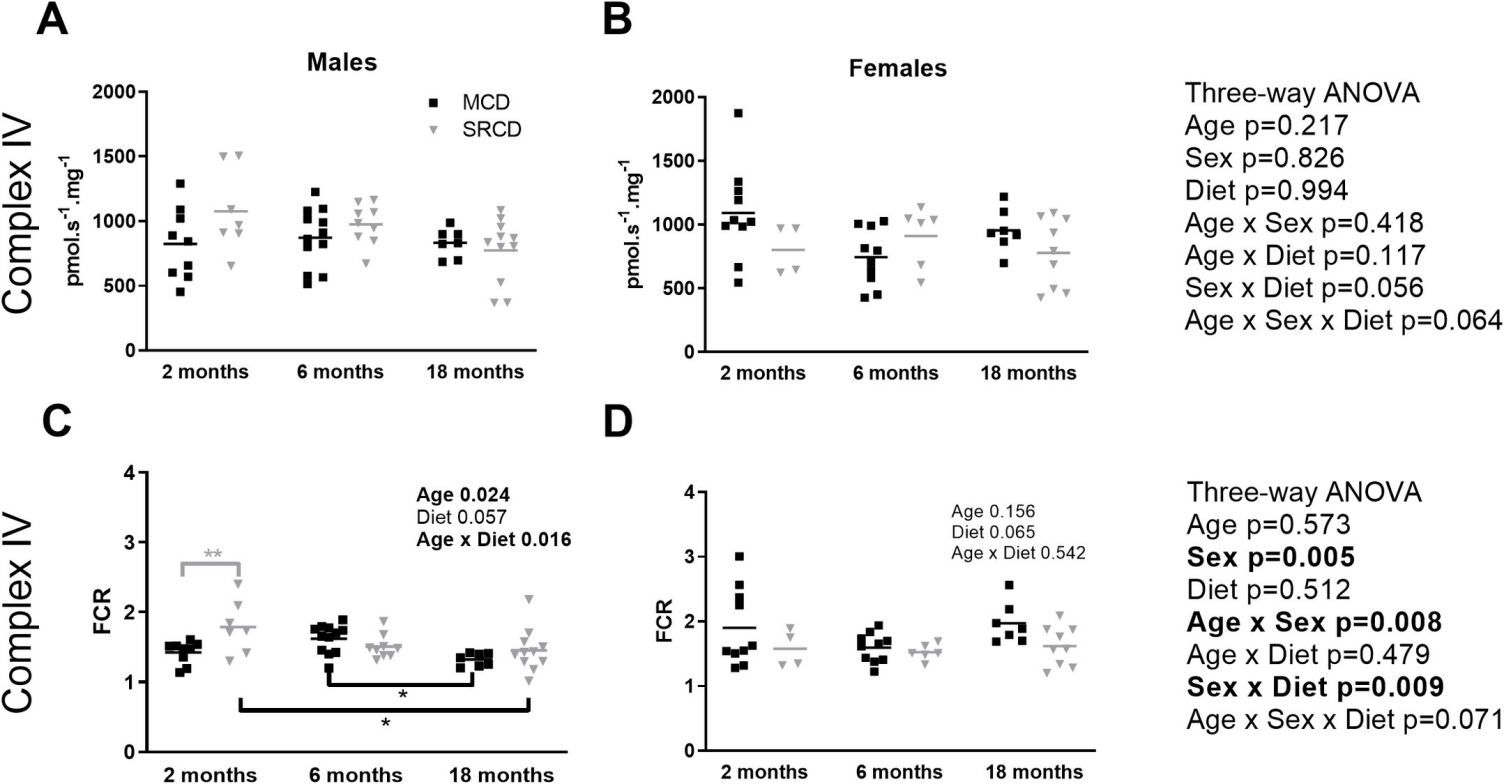
Complex IV capacity in the Nile Grass rat heart throughout the age range. Complex IV activity in cardiac permeabilized fibers from males (left) or females (right) Nile Grass rats fed the Mazuri Chinchilla Diet (MCD group, black squares) and the Standard Rodent Chow Diet (SRCD, grey triangles), at three ages (2 months, 6 months, and 18 months). Respiratory capacity was expressed in flux per fiber mass (pmol s^-1^ mg^-1^ wet weight; A and B) or as a flux control ratio (FCR), over the maximal capacity with substrates feeding electrons into both the NADH (N-) and the Succinate (S-) pathways simultaneously (C and D). Dot plot indicates the individual data points and the median. Results of the three-way ANOVAs are on the right. When there was a significant effect or interaction with sex, two-way ANOVAs were performed on separate sexes (results indicated on individual panels). The square root transformations were applied to meet the assumption of the female two-way ANOVAs for the N-pathway. Data are presented without transformation. N = 3–7 animals per group, and n = 4–12 fiber preparations per group. Significant differences between dietary groups (on the top, grey) and between age groups (on the bottom, black) are indicated with *** for p<0.001, ** for p<0.01, * for p<0.05.

Complex IV activity, measured with ascorbate and TMPD feeding electrons into complex IV, showed no significant change with age, sex, or diet when expressed in flux per mass (Fig 4A and 4B). Howerver, the three-way ANOVA on the FCR showed a significant effect of sex (p = 0.005), as well as interactions between sex and age (p = 0.008) and between sex and diet (p = 0.009; Fig 4C and 4D). The changes in complex IV were restricted to the males (Fig 4C). The 2-month males showed a higher FCR for complex IV in the SRCD compared to the MCD group (p = 0.004), and it was followed by a decrease with age (p = 0.015 between 2 and 18 months of age; Fig 4C). In the MCD group, the FCR for complex IV started lower at 2 months, increased at 6 months (without reaching significance) and then decreased significantly from 6 to 18 months (p = 0.029) (Fig 4C). The females, in contrast, did not show any change in FCR for complex IV with age or diet (Fig 4D). Overall, FCR for complex IV is the only part of the ET-pathway that was affected by diet and age, and again only in the males.

### The females, but not the males, showed diet-specific adjustment in their capacity to oxidize long-chain fatty acid

**Fig 5:**
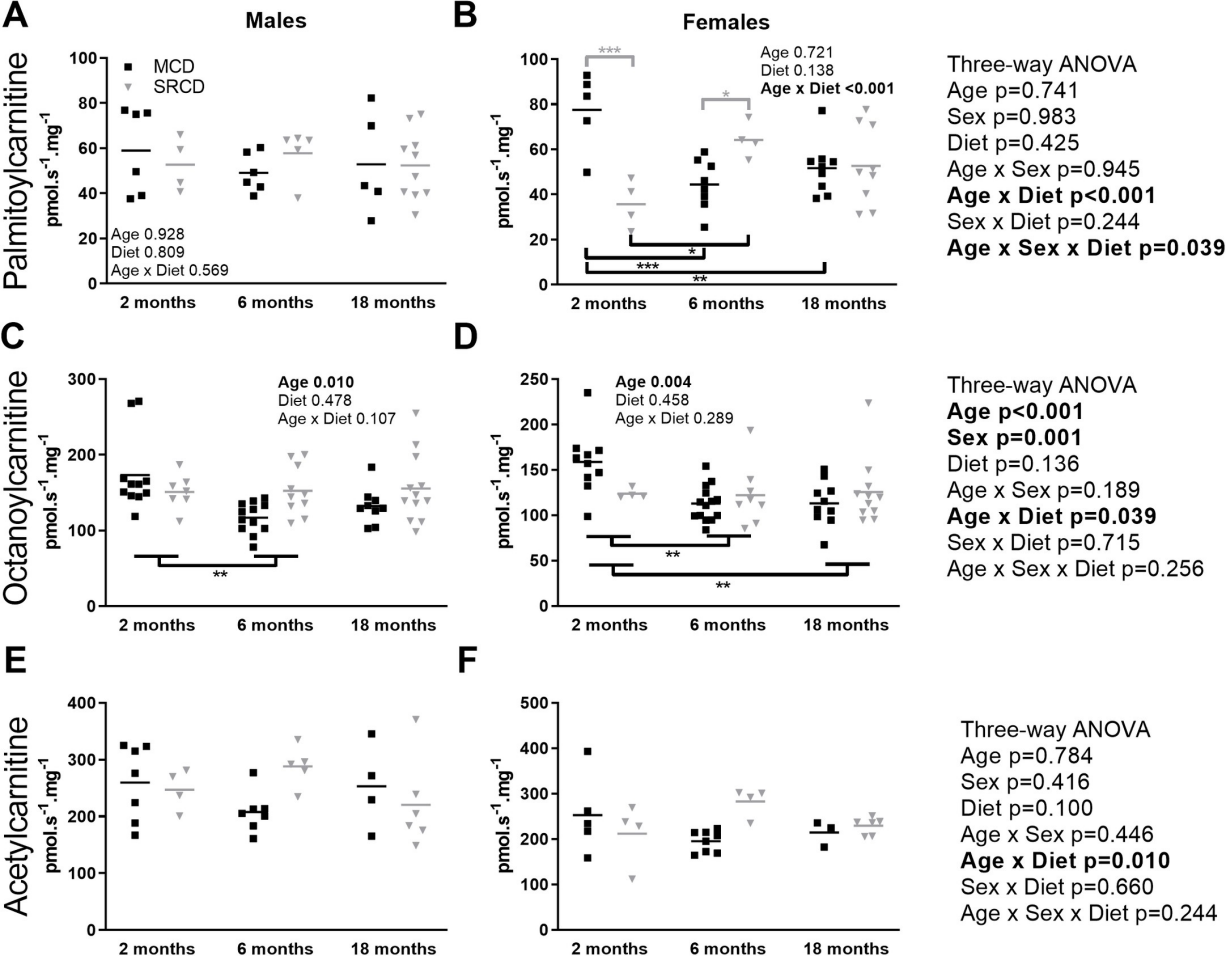
Fatty acid oxidation capacity in the heart of the Nile Grass rat throughout the age range. Oxidative phosphorylation (OXPHOS) capacity in the presence of fatty acids substrates in cardiac permeabilized fibers from males (left) and females (right) Nile Grass rats fed the Mazuri Chinchilla Diet (MCD group, black squares) or the Standard Rodent Chow Diet (SRCD, grey triangles) diets, at three ages (2 months, 6 months, and 18 months). OXPHOS capacity was expressed in flux per fiber mass (pmol s^-1^ mg^-1^ wet weight). The OXPHOS rate was determined in the presence of saturating ADP, cytochrome *c*, and three fatty acid substrates combinations: acetylcarnitine and malate (A, B), octanoylcarnitine and malate (C, D), and palmitoylcarnitine and malate (E, F). Dot plot indicates the individual data points and the median. The following transformations were applied to meet the assumption of the ANOVAs: reciprocal for two- and three-way ANOVAs for octanoylcarnitine data and three-way ANOVA for acetylcarnitine, and square root transformation for two-way ANOVA in males for octanoylcarnitine. N = 3–9 animals in the diet and age group, and n = 3–14 fiber preparations per group. Results of the three-way ANOVAs are on the right, and two-way ANOVAs on each panel. Significant differences between dietary groups (on the top, grey) and between age groups (on the bottom, black) are indicated with *** for p<0.001, ** for p<0.01, * for p<0.05.

We used three different protocols to measured fatty acid β-oxidation capacity in the presence of various substrates evaluating different enzymes and transporters (Fig 5; Table 1). Interestingly, the oxidation of the long-chain fatty acid palmitoylcarnitine does not vary with age or dietary group in the males (Fig 5A) but showed strong age- and diet-specific adjustments in the females (Fig 5B). The females fed on the SRCD increased their capacity to oxidize palmitoylcarnitine with age, whereas it decreased in the females of the MCD group. This is the reason for the differences between dietary groups at 2 (p<0.001) and 6 months (p = 0.024) of age in the females (Fig 3B).

The three-way ANOVA for oxidation of the medium-chain fatty acid octanoylcarnitine showed a clear effect of age (p<0.001) and sex (p = 0.001), as well as a significant interaction between age and diet (p = 0.039; Fig 5C and 5D, results on the right). When performing a two-way ANOVA on each sex, both males and females showed a decrease in oxidation of octanoylcarnitine with age (Fig 5C and 5D), but the effect was more pronounced in the females (Fig 5D) and was independent of the diet in both sexes (Fig 5C and 5D). The oxidation of acetylcarnitine (Fig 5E and 5F), which is the only substrate depending on the capacity of the acetyltransferase, was not affected by sex (p = 0.416), age (p = 0.784), or diet (p = 0.100). However, the SRCD group had a significantly higher capacity to oxidize acetylcarnitine with age and diet compared to the MCD group at 6 months of age (p<0.001 with pooled sexes).

### The effect of aging on mitochondrial coupling is independent of sex and is more pronounced in the SRCD group compared to the MCD group

**Fig 6:**
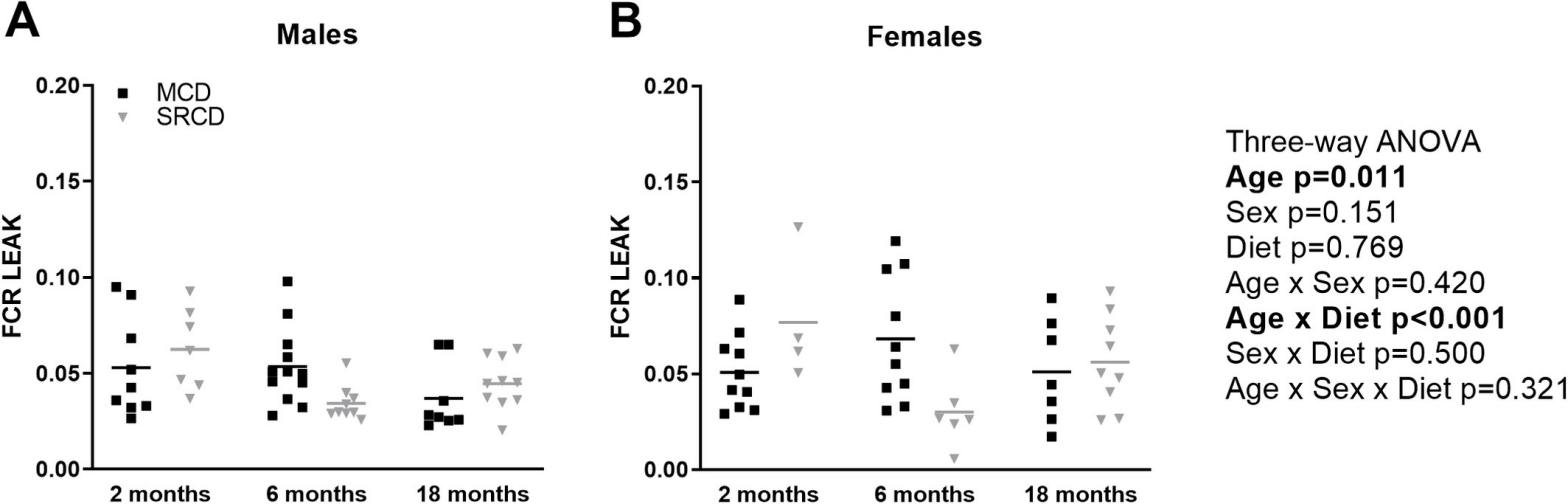
Mitochondrial coupling in the heart of the Nile Grass rat throughout the age range. LEAK respiration in permeabilized cardiac fibers from left ventricles from males (A) and females (B) Nile Grass rats fed the Mazuri Chinchilla Diet (MCD group, black squares) and the Standard Rodent Chow Diet (SRCD group, grey triangles) diets, at three ages (2 months, 6 months, and 18 months). The LEAK was determined in the presence of substrate feeding electrons into the NADH pathway (pyruvate and malate), and in the absence of ADP. LEAK respiration rate is expressed as FCR, over the maximal OXPHOS capacity with substrates feeding electrons into both the NADH and the Succinate pathways simultaneously. Dot plot indicates the individual data points and the median. The cube root transformations was applied to meet the assumption of the three-way ANOVA. N = 3–7 animals per group, and n = 4–12 fiber preparations per group.

The LEAK respiration in the presence of NADH-linked substrates was used to evaluate the coupling. It is defined as a state of mitochondrial respiration when oxygen flux mainly compensates for ion leaks in the absence of ATP synthesis. The FCR for LEAK was presented in Fig 6 as it is a more accurate way to evaluate the coupling of the OXPHOS process, independent from mitochondrial content and OXPHOS capacity [45]. A minimum value of 0.0 indicates a fully coupled system, while a value of 1.0 indicate a fully non-coupled system. The FCR for LEAK was not affected by sex (p = 0.151), but it was significantly affected by age (p = 0.011) and showed a significant interaction between age and diet (p<0.001; with a significant difference between dietary groups at 6 months of age). In general, the coupling decreases with age, but the effect appears earlier in the SRCD group (p<0.001 between 2 and 6 months; p = 0.013 between 6 and 18 months) compared to the MCD group (p = 0.024 between 6 and 18 months; Fig 6A and 6B).

### Mitochondrial outer membrane integrity

Exogenous cytochrome *c*, added after ADP, exerted a slight effect on OXPHOS capacity. It was expressed as the cytochrome *c* control factors (FCF*c*), indicating the control of respiration by externally added cytochrome *c* [45]. A value of 0.00 indicates no control of respiration by exogenous cytochrome *c* and complete integrity of the mitochondrial outer membrane. For males, the values were the following for the MCD group [2 months 0.08(0.05–0.14), 6 months 0.05(0.00–0.12), 18 months 0.08 (0.01–0.11)] and for the SRCD group [2 months 0.09 (0.05–0.19), 6 months 0.10(0.08–0.15), 18 months 0.06(0.00–0.09)]. For the females, the values were the following for the MCD group [2 months 0.10 (0.04–0.14), 6 months 0.05 (0.01–0.10), 18 months 0.09 (0.00–0.10)] and for the SRCD group [2 months 0.10 (0.00–0.18), 6 months 0.10 (0.07–0.14), 18 months 0.05 (0.00–0.11)]. Our values validate the integrity of mitochondrial membranes in the fiber preparation, with similar values to previous results in permeabilized cardiac fibers from rats [27, 46], mice [43], and humans [42]. The three-way ANOVA for the FCF*c* showed no effect of sex (p = 0.590), age (p = 0.094), or diet (p = 0.500). A significant interaction occurred only between age and diet (p<0.001). In the MCD group, membrane integrity was better preserved at 6 months compared to the 2-months age group (p = 0.019). In the SRCD group, the 18-months age group showed a better preservation of membrane integrity compared to both the 2- (p = 0.019) and 6-months (p = 0.002) age groups. The changes are most likely due to changes in mitochondrial membrane composition associated with age and diet [see 47, 48] that affect the sensitivity to the permeabilization procedure. To avoid any bias induced by change in membrane composition, OXPHOS capacity values used for comparison between age and groups are taken after addition of exogenous cytochrome *c*.

## Discussion

The role of mitochondria in insulin resistance and its progression to T2DM is still unclear and remains highly controversial. By using an innovative animal model, taking into account the sexual dimorphism, and including the progression throughout an age range, our study brings new insights to our understanding of the involvement of cardiac mitochondria in aging and T2DM. The gain in weight and BMI with age occurs at a faster rate in Nile Grass rats fed on an SRCD compared to an MCD. This is observed for both sexes. The animals also show hyperinsulinemia associated with both aging and SRCD, independent of sex. Interestingly, the females preserved their FBG within the control range throughout the 18-month period on the SRCD, while the males showed significant hyperglycemia starting at 6 months of age. It was previously suggested that the potential role of dietary fibers resides in controlling body weight and satiety [49], but because the female NRs do not show hyperglycemia, the apparition of T2DM in the NRs does not appear to be correlated with the weight gain. Our data also indicated that the MCD not only prevents hyperglycemia in the males but has a clear effect on age-related changes in cardiac mitochondria. These effects starts at an early age of 2 months, before the onset of hyperglycemia, and long before the mild sign of diastolic dysfunction is measured at 12 months [34]. The premature signs of mitochondrial variation points toward potentially important players in the progression of cardiac complications in aging and T2DM, and includes the mitochondrial content, the complex IV relative capacity, and the fatty acid oxidation patterns.

At 2 months, early in the progression of T2DM, the males fed the SRCD showed a higher cardiac CS activity compared to the MCD group. This was followed by a decline in CS activity. In males fed the MCD, the activity was stable throughout the age range. In the females no change in CS activity was measured with either age or diet. CS activity is known to strongly correlate with mitochondrial content when measured using transmission electron microscopy [50]. Our results indicate early changes, specific to the males, in mitochondrial content associated with the diet. Previous studies also reported an increase in mitochondrial content and/or biogenesis associated with high fat diet, alongside with detrimental enhancement of mitochondrial H_2_O_2_ emission [51–53] and insulin resistance [51, 54–57]. In contrast, the amount of cardiac mitochondria is either preserved in humans [13] or decreased in other animal models of T2DM such as the fructose-fed rat [17], the western-diet induced obese mice [58], and the db/db mouse [9]. The progression of the disease through aging in this study clearly helps to put some of the previous contradicting reports into perspective. The three time points included in our study are associated with different diet-related changes in the males; the mitochondrial content in the SRCD compared to the MCD group was higher at 2 months, similar at 6 months, and lower at 18 months. Because most studies focus on time points after hyperglycemia, the early increase in mitochondrial content observed might be missed and could be of meaningful relevance. Some studies suggest that a lower mitochondrial content represents an advantage against T2DM. Mice devoid of PGC-1α, a transcriptional co-activator and master regulator of mitochondrial biogenesis, exhibit a mild decrease in mitochondrial mass in most tissues. These mice also show increased insulin sensitivity, improved glucose tolerance, and do not develop T2DM when fed a high-fat diet [59, 60]. Similarly, muscle specific PGC-1α knockout mice have enhanced peripheral insulin sensitivity associated with reduced mitochondrial gene expression in muscle, but the global response to glucose in this model is negatively affected by a pancreatic defect [61].

In our study, the only group showing high prevalence to T2DM—the males fed the SRCD diet—is also the only one showing loss of cardiac mitochondrial content with aging. Similarly to the males fed the MCD, or the females on both diets, the standard rat fed a normal chow is not prone to develop T2DM, and show unaltered cardiac mitochondrial content during aging [27, 62–64]. The loss of mitochondrial content in the male NRs fed on the SRCD diet can be due to numerous metabolic changes associated with the development of T2DM, but it can be due the accelerated aging making the animals more prone to develop T2DM. A comprehensive metabolomics analysis in the ob/ob mouse heart supports aging as being the main factor causing mitochondrial defect. This study reveals that mitochondrial function is largely preserved in ob/ob mice fed with conventional rodent diet and deficiency in respiratory complexes I and II only appeared as the mice aged [65]. Up to now, the most successful lifestyle adjustments to reduce aging involves caloric, proteins or specific amino acid restrictions, alternate day fasting with similar calorie intake, [66] or physical activity [67]. In some studies, these interventions have also been related to improved mitochondrial content and function, reduced ROS production [reviewed by 68, 69], and improved insulin sensitivity [70–72]. In our study, without variation in food access, specific nutrient or exercise program, the males in the MCD group are protected against T2DM and age-related decrease in mitochondrial content as is observed in the SRCD group. The two diets contain similar amount of protein (19.9% for the SRCD and 21.6% for the MCD group), and of specific amino acids previously identified as able to affect mitochondrial function, i.e., leucine, methionine, threonine, valine, serine [66]. The SRCD diet contains the most amount of glutamic acid (4.68% compared to negligible amount), an amino acid identified as a promoting factor of longevity when given to yeast [66]. A common response of dietary changes or exercise is modification of the regulation by the sirtuins family of NAD^+^-dependent deacetylases [73]. The two main cardiac sirtuins, Sirt1 (in the nucleus) and Sirt3 (in the mitochondria), are activated upon caloric restriction, fasting or exercise, and inhibited by high-fat diet [66]. Increasing amount of studies support a primary role of sirtuins regulation in aging and age-related pathologies, including diabetes, cancer, and cardiovascular diseases [73]. Sirtuins activators, such as resveratrol, have been shown to extend lifespan [reviewed by 73] or health span [74] of numerous organisms, independent of weight loss [75]. Sirt1 is also involved in the protection of heart muscle from oxidative and hypertrophic stresses [see review by 76]. In addition, it protects the animals from developing diet- and age-induced insulin resistance and diabetes [74, 77–79]. A change in sirtuins regulation by diet is the most likely candidate to affect aging of cardiac mitochondria in the NRs, pointing toward a beneficial effect of the MCD on aging rather than specifically on T2DM.

Our study also pointed toward an interesting diet-specific pattern in the capacity of complex IV expressed relative to the maximal OXPHOS capacity (FCR) and is independent of the mitochondrial content. And these changes again occurred only in the males. The SRCD group shows a high complex IV capacity at 2 months which then decreases with age. The MCD group has a completely different pattern; it starts with a lower relative complex IV capacity at 2 months, followed by an increase for 6 months, and then a decrease back to initial capacity at 18 months. Complex IV plays an undoubtedly primordial role in aging and T2DM. Mice lacking the complex IV assembly protein Surf1 (Surf1-/-) display a dramatic reduction in complex IV activity in all tissues, but they also show an increase in energy expenditure, insulin sensitivity [80], and a modest extension of lifespan [81, 82]. The relationship between complex IV and insulin resistance is not a simple one. In the Sco2 knock-in/knock-out (KI/KO) mice, with one mutated allele of the Sco2 gene encoding a copper chaperone required for complex IV activity, complex IV activity is reduced by 20–60% in muscle; this is associated with an increase in insulin resistance [83, 84]. Two major differences in these two mice models can explain the opposite response to insulin. First, the mitochondrial respiration in several tissues of the Surf1-/- is unaffected by complex IV defect while the Sco2 KI/KO has a reduction in oxygen consumption, at least in the adipose tissue. In that regard, our Nile Grass rat model is more similar to the Surf1-/- mice. Our results show no significant changes in oxygen consumption of the OXPHOS pathways associated with the diet, suggesting an excess capacity of complex IV over pathway flux as previously measured in the mouse heart [43]. The second important difference between these animal models is that the Surf1-/- mice show an upregulation of the mitochondrial unfolded protein response pathway [85], whereas the Sco2 KI/KO do not [84]. Recent evidence suggests that the aging process emerges from programmed events occurring early in life and due to the inability to prevent metastable proteins from misfolding and aggregating [86]. The mitochondrial ET-pathway has been recently identified as a central regulator of the age-related decline of heat shock response and cytosolic proteostasis. A mild down-regulation of complex IV activity at an early age in the nematodes *Caenorhabditis elegans* results in an increased vitality later in life due to an increase in the sensitivity to protein misfolding through regulation of HSF-1 [87]. The pattern of complex IV activation in the NRs might be indicative of change in the cardiac aging process associated with the diet. An aging-activating switch may be triggered at an earlier time point for the males fed on SRCD. This idea is reinforced by the data in the females where an early increase in complex IV relative capacity in the SRCD group does not occur. Our findings warrant the exploration of a finely-tuned intervention aimed at modifying the mitochondria. Increasing the mitochondrial content or capacity might be detrimental at an early stage, but preventing the loss in mitochondrial capacity with aging might have a positive impact on the development of aging or aging-related diseases.

Our results identify variations in the ability to process fatty acid efficiently as a potential important factor that explains the high sensitivity of the male NRs to insulin resistance and T2DM. NRs are herbivorous murine rodents [88]; the SRCD is high in fat compared to their natural diet. Due to the energy requirements, the heart is an important consumer of fatty acids. This increased reliance on fatty acid for energy production has been shown in humans and animal models of T2DM [see review by 7]. Several studies have shown that the diet-induced activation of the fatty acid oxidation pathway can occur within days or three weeks [89–91], and is at least partly controlled by down-regulation of Sirt3 [92]. In insulin-resistant muscle from Zucker obese rats [93] or high-fat-fed rats [91], stimulation of the fatty acid oxidation pathway by overexpressing PGC-1α also increased insulin-stimulated glucose transport, suggesting an adaptive response to limit the damages. In the NRs, the males showed no diet-related adjustment in capacity to oxidize long- and medium-chain fatty acids. The lack of response of the fatty acid oxidation pathway while exposed to a high-fat diet can be a contributing factor to the NRs susceptibility to T2DM by enhancing intracellular lipid accumulation, a suggested major cause of insulin resistance [94, 95]. The females demonstrated a strong response to diet with the SRCD group, increasing its capacity to oxidize palmitoylcarnitine with age. However, in the MCD group, a reverse response with age was observed. Interestingly, the diet-specific response in the females is restricted to the long-chain fatty acids, without any diet-specific changes in the oxidation of the medium-chain fatty acid octanoylcarnitine. Dietary intake of long-chain fatty acids is known to play a causative role in insulin resistance [96]. When accumulated in the mitochondria, long-chain acylcarnitines inhibit pyruvate and lactate metabolism [97]; at higher concentration they inhibit OXPHOS and stimulate ROS production [98, 99]. Some studies also raised the possibility that the excess in long-chain acylcarnitines is able to act directly on the insulin signaling cascade through Akt phosphorylation [100, 101]. In our results, the inability of the male SRCD group to increase the oxidation of long-chain fatty acids may represent a strong disadvantage toward T2DM and aging.

A limitation in our study is the variation between the two commercial diets used. The selected diets have similar contents in carbohydrates and proteins, but not in fibers, fat, specific amino acids, vitamins, probiotics (only in the MCD), and potentially specific sugars (unknown for the MCD). In addition, the source of the fats, carbohydrates, and proteins are different, which introduced multiple variables and makes the data interpretation more complex [102]. A semi-purified diet would be more appropriate for this study; however, none have been developed to prevent the T2DM progression in the NR. In order to expand the potential of the NR as an animal model of T2DM, future studies should focus on the development of a semi-purified diet. Another limitation of our study is the sample size. The objective here was to include the diet, the sex and the time point to get a global idea of which mitochondrial changes are associated with aging and/or T2DM. It was, however, not sufficient to perform correlation between the mitochondrial function and either the FBG or the insulin level at specific time point. Furthermore, it did not allow to separate the animals between responders and non-responders, based on their insulin level. Future studies focusing on a more narrow age range would bring additional clarifications on the mitochondrial changes associated with age or playing a role in diabetes progression.

Our study addresses the sex-specific changes in cardiac mitochondrial function during the progression of aging and T2DM in the NRs. Since only the males develop T2DM on the SRCD, we can separate the changes associated with the diet (females) with the change associated with T2DM (males). We showed that changes in mitochondrial function associated with the diet are clearly sex-specific and these differences could likely explain the higher susceptibility of males developing T2DM compared to the females. The specific pattern of progression in mitochondrial content and complex IV relative activity likely indicates an accelerated cardiac aging process in the males compared to the females. Furthermore, the inability of the males to adjust their long-chain fatty acid oxidation makes them more prone to develop oxidative stress and insulin resistance in the heart. One of the challenges when studying variation of mitochondrial function in T2DM is to delineate if the variation in mitochondrial function are contributing to the cause or is only a consequence of the pathology. Our study shows early changes in mitochondrial function associated with the diet in the males, but not in females, pointing toward a role in the progression of the cardiovascular dysfunction associated with the disease. It also support the importance of understanding the whole progression of the disease, rather than at a single time point.
